# Life history of the Glanville fritillary butterfly in fragmented versus continuous landscapes

**DOI:** 10.1002/ece3.885

**Published:** 2013-11-22

**Authors:** Anne Duplouy, Suvi Ikonen, Ilkka Hanski

**Affiliations:** 1 Department of Biosciences, University of HelsinkiPO Box 65, Helsinki, FI-00014, Finland; 2 Lammi Biological StationLammi, FI-16900, Finland

**Keywords:** Dispersal, habitat fragmentation, flight capacity, larval growth, life-history evolution, male mating success, oviposition rate.

## Abstract

Habitat loss and fragmentation threaten the long-term viability of innumerable species of plants and animals. At the same time, habitat fragmentation may impose strong natural selection and lead to evolution of life histories with possible consequences for demographic dynamics. The Baltic populations of the Glanville fritillary butterfly (*Melitaea cinxia*) inhabit regions with highly fragmented habitat (networks of small dry meadows) as well as regions with extensive continuous habitat (calcareous alvar grasslands). Here, we report the results of common garden studies on butterflies originating from two highly fragmented landscapes (FL) in Finland and Sweden and from two continuous landscapes (CL) in Sweden and Estonia, conducted in a large outdoor cage (32 by 26 m) and in the laboratory. We investigated a comprehensive set of 51 life-history traits, including measures of larval growth and development, flight performance, and adult reproductive behavior. Seventeen of the 51 traits showed a significant difference between fragmented versus CL. Most notably, the growth rate of postdiapause larvae and several measures of flight capacity, including flight metabolic rate, were higher in butterflies from fragmented than CL. Females from CL had shorter intervals between consecutive egg clutches and somewhat higher life-time egg production, but shorter longevity, than females from FL. These results are likely to reflect the constant opportunities for oviposition in females living in continuous habitats, while the more dispersive females from FL allocate more resources to dispersal capacity at the cost of egg maturation rate. This study supports theoretical predictions about small population sizes and high rate of population turnover in fragmented habitats selecting for increased rate of dispersal, but the results also indicate that many other life-history traits apart from dispersal are affected by the degree of habitat fragmentation.

## Introduction

Habitat loss and fragmentation have caused the extinction of innumerable populations (Hughes et al. 1997; Hanski 2005). In the case of populations and metapopulations that have persisted, habitat loss and fragmentation may have altered natural selection on life-history traits and resulted in microevolutionary changes (Taylor and Merriam 1995; Thomas et al. 1998; Hendry and Kinnison 2001; Ronce and Olivieri 2004). Increasing numbers of researchers have concluded that fast (contemporary) evolutionary changes are more frequent than previously thought (Saccheri and Hanski 2006; Pelletier et al. 2009; Schoener 2011), and fast microevolution is especially likely to occur in changing environments, such as environments experiencing loss and fragmentation of habitat for the focal species (Hanski 2012b). Evolutionary changes may subsequently influence ecological dynamics, leading to reciprocal eco-evolutionary dynamics, which may in turn affect the risk of extinction (an example in Heino and Hanski 2001). Given the magnitude of anthropogenic habitat conversion in many biomes (Millennium Ecosystem Assessment 2005; Barnosky et al. 2011), it is imperative to develop a better understanding of the evolutionary as well as the ecological consequences of habitat loss and fragmentation.

Several theoretical and empirical studies have examined the evolution of dispersal in relation to habitat fragmentation (reviewed by Clobert et al. 2012). For instance, speckled wood butterflies (*Pararge aegeria*) originating from more continuous woodland habitats are more dispersive than butterflies from fragmented agricultural landscapes, where the species occurs in hedgerows (Merckx et al. 2003). Working on the bog fritillary butterfly (*Proclossiana eunomia*) in four landscapes ranging from a continuous to a highly fragmented landscape, Schtickzelle et al. (2006) found a significant decrease in dispersal propensity (emigration rate) with increasing fragmentation. In the plant *Crepis sancta,* dispersal capacity is lower in a fragmented city population than in a more continuous population in the countryside (Dornier and Cheptou 2012). In contrast, the Glanville fritillary butterfly (*Melitaea cinxia*) was inferred to be more dispersive in the more fragmented parts of a large network of 4000 meadows (Hanski 2011). These contradictory empirical results are paralleled by conflicting theoretical predictions. For instance, Heino and Hanski (2001) predicted increased dispersal rate with increasing habitat fragmentation, while Travis and Dytham (1999) predicted the opposite. The reason for inconsistent model predictions is likely to be the large number of factors that influence the evolution of dispersal (Matthysen 2012; Starrfelt and Kokko 2012) and the fact that different models typically consider different subsets of these factors. In particular, assumptions about the cost of dispersal, temporal stability of populations, and the availability of unoccupied habitat will greatly affect model predictions (Hanski and Mononen 2011). One additional complication is that habitat fragmentation may lead to evolutionary changes in other life-history traits apart from dispersal, which may in turn interact with the evolution of dispersal (Ronce and Clobert 2012). For instance, shorter life span (Kirchner and Roy 1999) and larger reproductive effort (Ronce and Olivieri 1997) may be selected for in metapopulations in fragmented habitats.

Empirical studies on the ecological and evolutionary consequences of habitat fragmentation would be most informative if studies would be conducted by comparing representative samples of individuals originating from landscapes with dissimilar degree of fragmentation. This has been rarely achieved; the studies by Schtickzelle et al. (2006) on the bog fritillary butterfly and by Cheptou and Dornier (2012) on the plant *C. sancta* are two examples. Instead, most empirical studies have compared local populations inhabiting particular habitat patches in different landscapes rather than representative metapopulations from different landscapes with different degrees of fragmentation. In particular, researchers have compared dispersal rate of individuals in isolated versus well-connected populations (Cody and Overton 1996; Malcolm et al. 2002; Fresnillo and Ehlers 2008; Lepais et al. 2010; Hanski and Mononen 2011) and in new versus old populations (Thomas et al. 2001; Hanski et al. 2006; Ovaskainen et al. 2008; Honnay and Jacquemyn 2012). Such comparisons are informative for the effects of fragmentation, as the frequency of new and isolated populations can be expected to be higher in fragmented than continuous landscapes (CL). Nonetheless, it would be preferable to conduct empirical studies at the landscape level, as local populations may exhibit specific characteristic not representative of the whole metapopulation (Hanski et al. 2006; Ovaskainen et al. 2008).

Here, we compare the life histories of four regional populations of the Glanville fritillary butterfly (*M. cinxia*) inhabiting two highly fragmented landscapes (FL) versus two CL in northern Europe. In the past, the Glanville fritillary probably inhabited extensive grasslands across its geographical range in Europe and Asia (Hanski 1999), but presently, it occurs in regions with very different degree of habitat fragmentation. The Åland Islands in Finland and the Uppland coastal area in Sweden represent two highly FL, in which individual habitat fragments (dry meadows) are small, typically ≪1 ha (Ojanen et al. 2013). In contrast, on the large Baltic islands of Öland in Sweden and Saaremaa in Estonia, habitat for the Glanville fritillary occurs in much larger continuous areas, calcareous “alvar” grasslands, often exceeding 100 ha in area (Helm et al. 2006). Comparing more than two fragmented versus two CL would be preferable, but genuine replication at the landscape level is difficult for most wild species, and in the present case, no other well-known populations were available in the Baltic Sea region. Here, a particular complication is the slightly more northern location of the FL (Åland and Uppland) in the Baltic area, but there are reasons to conclude that the difference in the degree of habitat fragmentation between the regions is more consequential for life-history evolution (Discussion). We conducted common garden experiments in a large outdoor population cage and in the laboratory, using butterflies that had been sampled either as pre-diapause larvae in the field or were reared in the laboratory. We characterized life histories by measuring a large number of larval and adult traits. In the case of traits that were measured on individuals sampled in the field prior to winter diapause, the results may reflect environmental as well as genetic effects, but in any case, possible associations with landscape structure are of interest. In the case of traits measured on laboratory-reared individuals, we can more confidentially assign any associations with landscape structure to genetic effects. We test the prediction from models parameterized for the Glanville fritillary (Heino and Hanski 2001; Zheng et al. 2009; Hanski and Mononen 2011) that dispersal rate increases with habitat fragmentation, which contrasts the common notion that increasing cost of dispersal in FL typically selects for reduced dispersal (Dytham and Travis 2012). There are no comparable model predictions for any other life-history traits, and here, we test whether there are significant differences between the fragmented versus CL, which would imply dissimilar selection pressures in the contrasting landscape types.

## Materials and Methods

### Study populations

The Glanville fritillary (*M. cinxia* L.) is widely distributed from Western Europe to Asia and North Africa. The species' habitat consists of dry meadows and grasslands with one or more of the larval host plant species, typically *Plantago lanceolata* and *Veronica spicata* in northern Europe. For a detailed description of the ecology and life history of the Glanville fritillary see Nieminen et al. (2004), and for demographic and eco-evolutionary dynamics see Hanski (1999, 2011).

**Figure 1:**
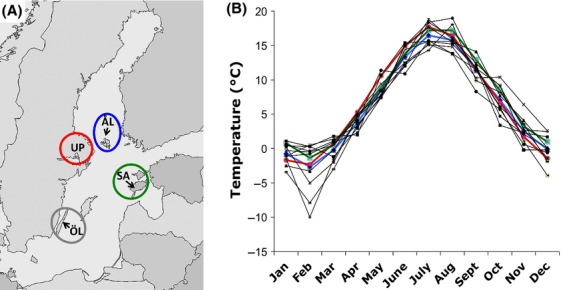
(A) Map of the Baltic Sea region with the populations of the Glanville fritillary included in the present study: Åland Islands (ÅL), Uppland coastal region (UP), Öland (ÖL), and Saaremaa (SA). (B) Average monthly temperatures between 1992 and 2001 in ÅL (Blue), UP (Red), and SA (Green). The dashed lines give the average monthly temperatures recorded in ÅL.

We collected study material from four large regional populations in the Baltic Sea region, including the well-studied metapopulation in the Åland Islands (ÅL) in Finland, the Swedish east-coast population in Uppland (UP), and the populations on the large Swedish island of Öland (ÖL) and the large Estonian island of Saaremaa (SA) (Fig. 1A). The effective size of the ÅL metapopulation is roughly *N*_e_ ≈ 10,000, based on long-term census data (Hanski 2012b). Based on their areas, the other regions have comparable large regional populations. ÅL and UP have highly FL, with most habitat patches smaller than one hectare (in ÅL the average size is 0.17 ha; Nieminen et al. 2004). The bedrock in these regions is made up of volcanic rocks formed more than 1700 million years ago. (Katrantsiotis 2013). Following land up-lift since the last glacial period, the landscape is a small-scale mosaic of granite outcrops and intervening areas with sedimentary deposits supporting forests and presently small cultivated areas. In ÅL, some loss of seminatural meadows may have occurred in the past 100 years (Ojanen et al. 2013), but in any case, the landscapes in ÅL and UP have always been highly fragmented. In contrast, the habitat in ÖL and SA occurs in large continuous calcareous grasslands (alvars), often extending across several hundred hectares. In SA, the area of alvar grasslands has declined, with average area of individual (contiguous) alvars decreasing from 3.6 km^2^ in the 1930s to 20 ha in 2000 (Helm et al. 2006), which nonetheless is still two orders of magnitude greater than the average meadow size in ÅL. There is no detailed mapping of habitat patches at large spatial scale in UP and ÖL, but field observations indicate that landscape structure in these regions is very similar to that in ÅL and SA, respectively, and hence, the contrast between fragmented and CL is very clear.

According to monthly average temperature records for 1992–2001, ÅL, ÖL and SA have similar climatic conditions, with average temperatures approaching 20°C during summer and dropping below zero in winter (Fig. 1B). Note that the average temperatures for ÖL and SA are well within the range of yearly variation in ÅL during the 10-year period (Fig. 1B). Nonetheless, some slight differences in climatic conditions may be present due to the slightly more northern latitude of ÅL and UP (∼60°N) than SA (∼58°N) and ÖL (∼57°N). The larval host plants *P. lanceolata* and *V. spicata* are present in all study areas. All four regional populations of the Glanville fritillary used in this study belong to the eastern clade (Wahlberg and Saccheri 2007) based on genome-wide SNP data (P. Somervuo, J. Kvist, S. Ikonen, P. Auvinen, L. Paulin, P. Koskinen, L. Holm, M. Taipale, A. Duplouy, A. Ruokolainen, S. Saarnio, J. Sirén, J. Kohonen, J. Corander, M. Frilander, V. Ahola, I. Hanski, unpubl. data) with some admixture with the central European clade in ÖL (based on COI haplotypes; Genbank #KC465909-KC465917).

The Glanville fritillary lays clutches of 150–200 eggs, and larvae remain gregarious during most of their development (Nieminen et al. 2004). Material for the present experiments was collected from the field as prediapause larvae in two occasions, in September 2006 (G0-2006) and in September 2009 (G0-2009) (“G0” referring to generation zero; Table 1). The larval families that were sampled were widely distributed across each region, roughly within area of 10 by 10 km, and hence, it is fair to assume that the larval families were unrelated to each other and representative of the respective regional populations. The sampling included 34 families in 2006 (9 ÅL, 12 SA and 13 UP) and 200 families in 2009 (50 in each population). The G0-2006 prediapause individuals were reared in family groups, while the G0-2009 material was reared in four mixed groups of 50 unrelated individuals, each originating from a different larval family. We intended to use only one such mixed group for the cage experiment (below), but as some mortality occurred during the larval period, we supplemented the material with some extra individuals from the other groups. We later estimated the relatedness of the experimental individuals in the G0-2009 material based on their genetic similarity (SNPs, data not shown). We found potential sibling relationships between eight pairs of individuals from ÅL, six pairs and one triplet from ÖL, 15 pairs and one triplet from SA and ten pairs from UP, leaving a total of 27, 27, 42, and 32 independent families, respectively.

**Table 1:** Sample sizes in the different study materials

Landscape	Fragmented (FL)				Continuous (CL)			
		
Population	Åland (ÅL)		Uppland (UP)		Öland (ÖL)		Saaremaa (SA)	
				
Sex	♂	♀	♂	♀	♂	♀	♂	♀
G0-2006 (larval stage)	16	15	18	29	–	–	22	15
G0-2009 (larval and adult stages)	21	14	26	18	22	17	27	31
G1-2009								
Flight metabolic rate	12	9	10	8	4	4	13	11
Adult morphology	26	20	22	9	10	5	22	11
Egg weight	–	29	–	16	–	4	–	15

### Larval and pupal development

The field-collected larvae over-wintered in the laboratory in incubators in the constant temperature of 3°C. Following the end of diapause in April, the larvae were reared indoors under common garden conditions: 12:12 L/D and 25/15°C day/night temperature, respectively. Larvae were fed on excess of cut leaves of *P. lanceolata*. G0-2006 postdiapause larvae were first reared in small family groups and individually from the seventh (final) larval instar onward in small containers (4 cm diameter, 7 cm height). G0-2009 postdiapause larvae were reared individually from the fifth instar (end of diapause) onward. Laboratory-reared offspring of the G0-2009 butterflies, denoted as G1-2009 (the first laboratory generation), were reared in small family groups under the same conditions as the parental generation.

The G0-2006 larvae were weighed individually in the beginning of the seventh instar. For the G0-2009 material, we recorded the dates of the fifth, sixth, and seventh instar molts as well as the weight of each caterpillar at the beginning of each instar. We calculated the time of postdiapause development as the number of days between the fifth instar molt and pupation. Similarly, the development time of each postdiapause instar is the number of days between two consecutive molts. We recorded the pupal weight and pupal development time. The relative larval growth rate (RGR) was calculated as the difference between the pupal weight and the fifth instar larval weight, divided by the fifth instar weight and by the development time.

### Outdoor cage experiment

Butterflies were marked individually by writing a number on the underside of the hindwing. Marking took place on the day following eclosion, thus ensuring that all butterflies had fully extended and dry wings prior to handling. Due to a problem with the timing of larval development, the experiment on the adults of the G0-2006 material largely failed; hence, we only analyzed larval traits for this material. Table 1 summarizes the sample size for the different experiments.

**Figure 2:**
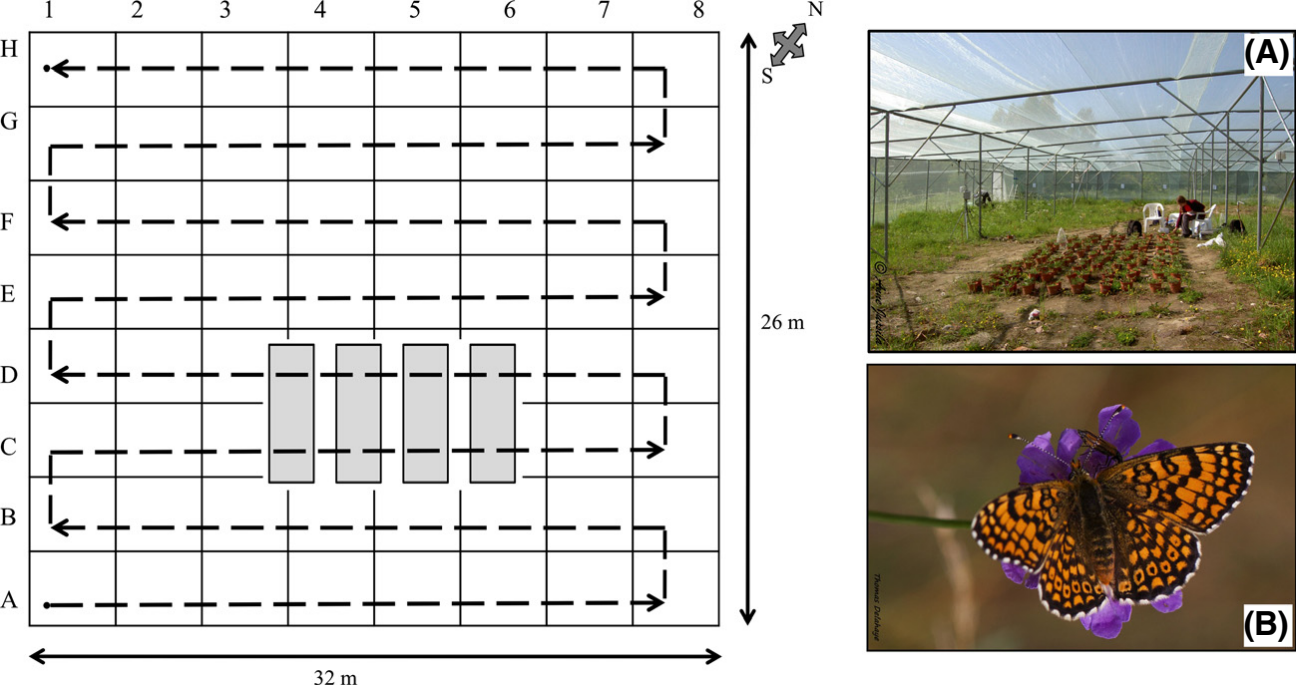
Schematic representation of the large outdoor population cage, with the transect surveyed five times a day (dashed line), the potted host plants in the central part of the cage (gray areas), and the grid division of the cage. (A) An inside view of the cage with an assistant monitoring the 200 potted host plants (© A. Jussila). (B) Close-up of the Glanville fritillary butterfly (*Melitaea cinxia*) (© T. Delahaye).

G0-2009 butterflies were released 24 h after their eclosion into a large outdoor cage in June 2010 (32 × 26 × 3 m, Fig. 2). The cage covered a dry meadow rich in flowering plants and closely resembling the natural habitat of the Glanville fritillary (Hanski et al. 2006; Saastamoinen 2008). The central part of the cage had 200 potted host plants, 100 individuals of *P. lanceola* and *V. spicata* each, individually labeled and placed within a regular grid within an area of 3.9 × 9.5 m (Fig. 2). These plants were provided for females to oviposit on, while all other host plants had been removed from the cage. After the 10th sunny day of the experiment, each remaining female butterfly was placed singly into a small cage of 10 (radius) × 20 cm, topping a potted host plant, to allow the female to continue ovipositing. Remaining males were similarly collected and kept in small groups (<15 individuals) in cages of 40 (radius) × 50 cm. The butterflies were kept in the small cages for the rest of their life to allow the measurement of remaining ovipositions and longevity, while minimizing the loss of dead butterflies that are difficult to recover from the outdoor cage. The dead butterflies were sampled for DNA for further phenotype–genotype association studies (not reported here). Four assistants worked continuously in the cage during the butterflies' active daily period, from 8 am to 6 pm, to collect data.

#### Adult behaviors

The cage was surveyed for marked butterflies five times a day (Fig. 2) every second hour, starting at 9, 11, 13, 15, and 17 h, weather permitting. We recorded the position in the cage and the behavior of each butterfly encountered as follow:

- Basking: butterfly steady on the ground, on a plant or on the cage wall, wings open.
- Resting: butterfly steady on the ground, on a plant or on the cage wall, wings closed.
- No movement: butterfly resting or basking.
- Flying: butterfly flying.
- Mating: butterfly paired with a sexual partner.
- Laying: female ovipositing on a host plant.

Mating pairs were additionally searched continuously throughout the cage during the experiment. For each mating pair encountered, the identity of both the male and the female, their position in the cage as well as the time of the day and the ambient air temperature were recorded. The male mating success of butterflies originating from the different populations was compared with a null hypothesis based on random mating given the total numbers of females and males from each population present in the cage in each day (empirical data compared with 10,000 randomizations).

#### Mobility

For each butterfly, we regressed the total number of grid cells in the cage (Fig. 2) in which the butterfly was observed against the respective total number of observations during the transect surveys. As the age may influence butterflies' mobility, we measured mobility over two periods, first over the first 3 days of life, and second over the butterflies' entire life span. The residual from the regression line was used as a measure of mobility for each butterfly (Hanski et al. 2006). The distance covered by a butterfly was calculated as the cumulative distance between all observations. We regressed the distance covered by the butterfly against its life span and used the residual from the regression line as a measure of life-time independent movement distance in the cage.

#### Longevity

The life span of each individual was defined as the time between eclosion and natural death. If the death was not recorded and the butterfly's body was lost, the date of the last observation alive in the cage was used as the date of death. This method gives an accurate result, as we conducted five surveys per day and the probability of detecting a butterfly that was alive in each survey was ∼0.5.

#### Oviposition

The 200 potted host plants were constantly monitored between 8 am and 6 pm to record all ovipositions. For each oviposition, we recorded the identity of the female, the plant species, the time of the day, and the temperature at the start of the oviposition. The positions of individual plant pots in the grid of plants were randomized in each evening. After the oviposition, the leaf with the egg clutch was detached, placed on a petri dish, and brought to the laboratory for rearing in an incubator. After 3 days, eggs were counted to determine the clutch size. Lifetime-corrected number of eggs for each female was calculated as the total number of eggs laid by the female divided by its life span. The oviposition rate was defined as the total number of ovipositions (egg clutches) divided by the life span. The preference of a female to oviposit on *P. lanceolata* was calculated as the fraction of clutches laid on this host plant species.

The G1-2009 butterflies, the laboratory-reared offspring of the G0-2009 butterflies, were used for three laboratory experiments described below:

#### Egg weight

The G1-2009 females from each population were mated in small cages with a male from the same population but from a different family, thus avoiding outbreeding and inbreeding. Mated females were placed individually in small cages with a potted host plant (*V. spicata*) on which they oviposited. Each egg clutch was collected singly onto a petri dish. Clutches were weighed, and the eggs were counted 3 days after oviposition. The average egg weight was calculated by dividing the weight of the clutch by the number of eggs.

#### Adult morphology

Ten random G1-2009 butterflies (five males and five females) from each family were frozen on the day after eclosion. After dissection, abdomen and thorax (head discarded) were dried for 24 h at 60°C and weighed. Wings were scanned and the right forewing areas were measured as described by Mattila et al. (2012). If the right forewing was damaged, the left forewing was used instead. Thorax-wing load was calculated by dividing the thorax dry weight by the wing area (mg/mm^2^), and total wing load by dividing thorax plus abdomen dry weights by the large wing area.

#### Metabolic rate

The flight metabolic rate (FMR) of four G1-2009 adults (two males and two females) from each family was measured as described by Niitepõld et al. (2009). In brief, a butterfly was placed into a hermetic chamber through which CO_2_-free air was pumped and was forced to fly continuously for 10 min. The CO_2_ concentration inside the chamber was recorded for more than 12 min, including the 10 min of forced flight and two mins of inactivity prior to the forced flight. Four measures were calculated: (1) The highest level of CO_2_ produced during the experiment (CO_2_ peak; mL/h); (2) the total volume of CO_2_ (mL) produced during the experiment; (3) the total volume of CO_2_ (mL) produced during the last 5 min of the experiment (reflecting flight endurance); and (4) the average resting metabolic rate (RMR; mL/h), calculated as the rate of CO_2_ emitted during the 60 sec prior to the start of the forced flight period. Temperature in the metabolic chamber was regulated at 30°C (Niitepõld et al. 2009).

Many of the above traits are correlated with each other. We nonetheless present results on a large number of traits (Appendix S1) as they may be of value for comparisons with other species and studies. To aid interpretation of results on many correlated traits, we have also performed multivariate analyses of several sets of traits (Appendix S2).

### Online resources

The data supporting the research have been archived in the public archive “Dryad” under the provisional data identifier: doi: 10.5061/dryad.2v3p5.

### Statistical analyses

Statistical analyses were carried out using R64 (R Development Core Team 2009). All data were tested for normality and transformed prior analysis when appropriate. We used log transformation to normalize data on the age at first oviposition, the age at first mating, and the first oviposition event, and ArcSin transformation on proportions and probabilities. We analyzed larval, pupal, and adult traits (G0-2006 and G0-2009 samples) using ANOVAs with sex, population, and landscape type (FL or CL) as fixed factors, with population (ÅL, ÖL, SA, and UP) nested within landscape. While analyzing the larval developmental time and seventh instar weight, the year (2006 or 2009) was included as another fixed factor. We tested for the effects of pupal weight on lifetime-corrected number of eggs laid, of the clutch rank on clutch size, and of the clutch size and clutch rank on the length of ovipositing the clutch and on the period between two consecutive ovipositions. Longevity was analyzed with a general linear model with inverse link function. Traits related to egg weight, metabolic rates, wing size, and body parts (G1-2009) were tested using a mixed model, with family nested in population as a random factor and landscape type and sex as fixed factors. When appropriate, we calculated pairwise comparisons using the post hoc Tukey HSD test and corrected for multiple testing by applying the Bonferroni's adjustment (*α* = 0.05).

We used R64 to calculate four principal component analyses (PCA). In the first analysis, we included larval, pupal, and adult traits (including flight behavior, mobility and longevity) shared by males and females. The second PCA included only the larval traits for both sexes, while the third and the fourth PCAs included only female traits, either all traits or adult traits only, respectively. We analyzed the principal components with ANOVAs, with landscape type and population nested within landscape type as explanatory factors, and sex in the PCA1 and PCA2.

## Results

We measured 51 traits characterizing larval development, adult flight capacity, behavior, and reproductive performance (Table A1). Of the 51 traits, 17 traits showed a significant difference between the fragmented versus CL. These traits cluster into three main groups, namely traits characterizing larval weight and development, traits of FMR, and traits of adult reproduction. Below, we present the main results for each set of traits. Details are given in the Appendix S1 (Table A2) for traits with significant landscape effects and for traits that are discussed in the text.

### Larval and pupal development

**Figure 3:**
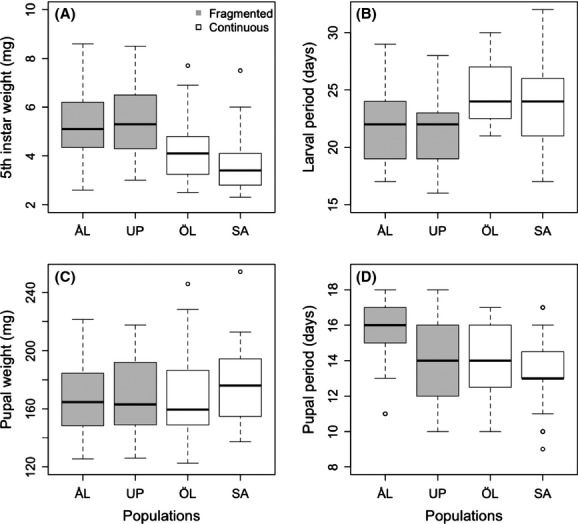
Larval and pupal development. (A) Weight of the fifth larval instar (mg), (B) larval period (days), (C) pupal weight (mg), and (D) pupal period (days) in Åland (ÅL), Öland (ÖL), Saaremaa (SA), and Uppland (UP) populations. Fragmented landscapes (FL) and continuous landscapes (CL) populations in gray and open boxes, respectively. Heavy horizontal lines represent median values.

There were significant differences in several larval traits between the FL and CL (Fig. 3). Larvae from FL populations were heavier than those from CL populations following the diapause at the fifth (Fig. 3A; 5.4 vs. 3.9 mg, respectively) and sixth instars, but at the seventh (final) instar the weights were similar. CL larvae compensated for their smaller initial size by having longer fifth instar and therefore longer postdiapause larval period (Fig. 3B), especially in males. There was a difference between the 2 years: the G0-2006 individuals had a significantly shorter larval period than the G0-2009 individuals (*P* = 2.2e-16).

Pupae from the two landscape types had similar weights (e.g., 167 mg in FL vs. 174 mg in CL populations in the G0-2009 material; Fig. 3C). FL individuals had longer pupal period (*P* = 0.0018), largely due to the slower pupal development of ÅL individuals (*P*_adj_ *=* 1.2e-4, Fig. 3D). The pupal period was significantly longer in males than females (*P* < 2.2e-16).

### Reproduction and longevity

Åland Islands males had significantly higher overall mating success in the outdoor cage than expected by random matings (*P* = 0.0028; Table 2). Both ÅL and UP males, hence males from FL, showed higher frequency of multiple matings than expected (*P* *=* 0.0016 and *P* *=* 4.0e-4, respectively), thus contrasting with males from CL with no excess of multiple matings. Intriguingly, ÅL butterflies showed a tendency toward assortative mating, with significantly more ÅL × ÅL matings than expected by chance (*P* *=* 6.0e-4, Table 2). There was no difference in the total number of matings between females from the different landscape types.

**Table 2:** Male mating success in the outdoor cage experiment (G0-2009 material, Table 1)

Landscape	Fragmented (FL)				Continuous (CL)			
		
	Åland (ÅL)		Uppland (UP)		Öland (ÖL)		Saaremaa (SA)	
				
Population	Obs	*P*	Obs	*P*	Obs	*P*	Obs	*P*
All matings	27 (>)	2.8e-3**	22	0.80	17	0.11	20	0.79
First mating	17	0.08	12	0.996	12	0.23	14	0.93
Multiple matings	10 (>)	1.6e-3**	10 (>)	4e-4***	5	0.12	6	0.15
Within-population matings	8 (>)	6e-4***	6	0.52	6	0.15	11	0.14

The *P*-values give the probability of the observed number of matings (Obs) being significantly different than expected by assuming random matings among the butterflies present in the cage. Results are based on 10,000 randomizations. “>” indicates that the observed number of matings is significantly greater than expected.

**P < 0.01, ***P < 0.001.

**Figure 4:**
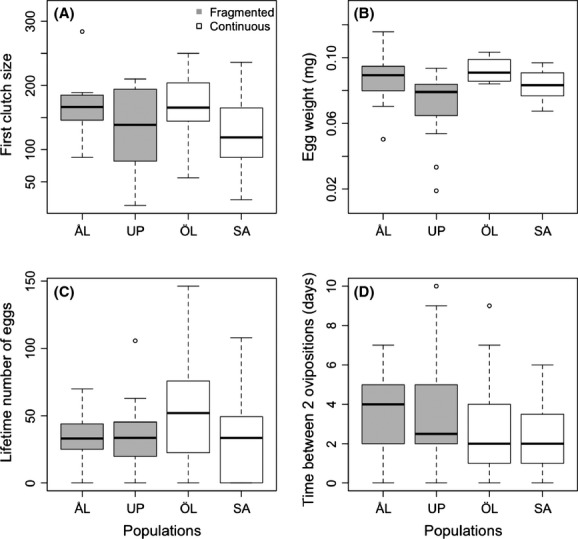
Ovipositions. (A) First clutch size, (B) egg weight (mg), (C) lifetime-corrected number of eggs laid (N/day), and (D) time between two consecutive ovipositions (days) in Åland (ÅL), Öland (ÖL), Saaremaa (SA), and Uppland (UP) populations. Fragmented landscape (FL) and continuous landscape (CL) populations in gray and open boxes, respectively. Heavy horizontal lines represent median values.

Egg-clutch size (corrected for female's pupal weight) decreased with the rank number of the clutch (*P* *=* 0.0013) and therefore with the age of the female (Table A2). There were no differences in average egg weight between the landscape types nor populations. We did not detect any trade-off between egg weight and clutch size, rather the opposite, as egg weight and clutch size are positively correlated especially when considering only the first clutches (Fig. 4A,B).

Females from FL were older (4 days on average) on their first oviposition than CL females (3.3 days; *P* = 0.013). The time between two consecutive clutches increased with the size of the present clutch (*P* = 1.23e-11) and with clutch rank (and hence with female age, *P* *=* 1.77e-5), and the time between two clutches was significantly longer in FL than CL females (*P* = 6.17e-5, Fig. 4D). There was no landscape type nor population effect on the oviposition rate, nor on the lifetime-corrected number of eggs laid (Fig. 4C), and there was no significant effect of population, landscape type or sex on adult longevity (Appendix S1, Table A2).

### Flight metabolism, behavior, and morphology

**Figure 5:**
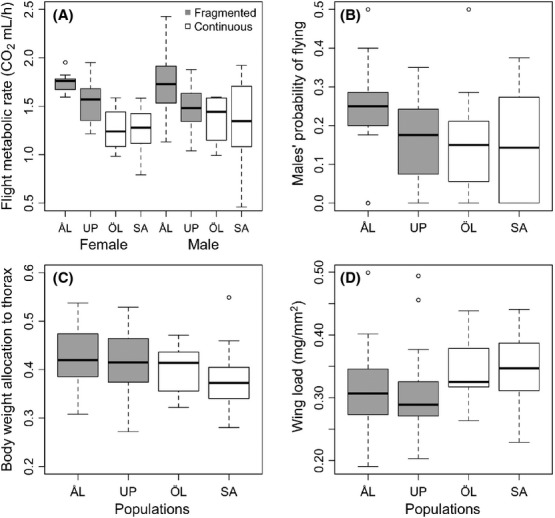
Flight behavior, metabolism, and morphology. (A) Peak flight metabolic rate (CO_2_ production, mL/h, corrected for pupal weight) during forced flight in females and males, (B) Probability of flying in males in the outdoor population cage, (C) body weight allocation to thorax and (D) wing load ratio (mg/mm^2^) in Åland (ÅL), Öland (ÖL), Saaremaa (SA), and Uppland (UP) populations. Fragmented landscape (FL) and continuous landscape (CL) populations in gray and open boxes. Heavy horizontal lines represent median values.

We used residuals from the regression of metabolic rate against pupal weight to correct for the effect of body size on metabolic rate. Weight-corrected RMRs were similar across populations, landscape types, and sexes. In contrast, all weight-corrected measures of FMR were significantly different between the landscapes types: CL butterflies exhibited lower FMRs than FL butterflies (Fig. 5A and Appendix S1, Table A2).

In the outdoor cage, we recorded different behaviors related to butterflies' flight and mobility. Males from FL flew more frequently than females, but there was no such difference in CL (sex–landscape interaction, *P*_adj_ = 0.016, Table A2). Males' mobility was higher than that of females in all populations, but there were no differences between populations nor landscape types in the frequency of resting. The mobility of young butterflies (<3 days old) was similar between the sexes, but young FL butterflies tended to be less mobile than young CL butterflies (*P* = 0.028; Appendix S1, Table A2).

In the laboratory-reared material (G1-2009), there were no differences in the weights of body parts nor in the allocation to thorax between FL and CL butterflies (Fig. 5C). As expected, females invested more of total weight in abdomen and males invested more in thorax (*P* < 1.0e-4). There were no differences in wing area among populations nor among landscape types, but there was a significant difference in wing loading ratio between the landscape types, with smaller values in FL than CL butterflies (0.31 vs. 0.34 mg/mm^2^; Fig. 5D).

### Multivariate analyses

**Figure 6:**
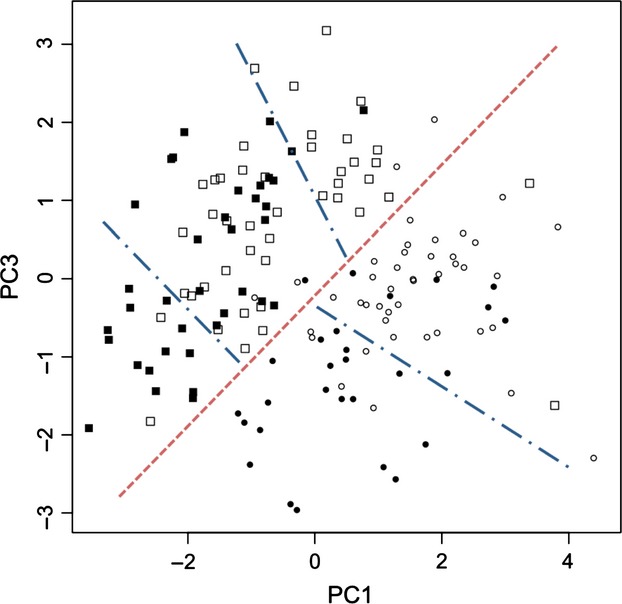
Principal component analysis of larval and pupal traits. The two principal components PC1 and PC3 from the third principal component analyses in Table 3. Squares for males, circles for females, open symbols for continuous landscapes (CL) and closed symbols for fragmented landscapes (FL). The dashed lines were drawn by eye to ease visualization. CL and FL individuals are well differentiated in females but there is more overlap in males.

The interpretation of the above results is potentially complicated by correlations between the variables. We hence run PCA for several data sets, including either both larval and adult traits or only larval traits, and including both sexes or females only (Table 3). In all analyses, there were highly significant differences between landscape types in PC1, but no large differences between the populations nested within landscape type (Table 3; full results in Table 4 and Appendix S2, Table B). In the analyses including both sexes, the first principal component showed additionally highly significant differences between the sexes, both in analyses based on larval and pupal traits only and in analyses based on larval and adult traits (PCA2 and PCA1, respectively). Figure 6 illustrates the strong discrimination between the sexes and the landscape types in the analysis for larval traits. Note that especially in females, the contrast between butterflies from the two landscape types is very clear. Examining correlations between the PCs and the original variables indicates that the largest differences between the landscape types are in postdiapause larval weight and the length of the pupal period (greater in FL), consistent with the analyses of individual traits. The periods of larval instars are short in FL, which contributes to the short larval development time (Fig. 3B).

**Table 3:** First principal components from four principal component analyses (PCAs). PCA1 and PCA2 include both male and female data (G0-2009), with and without adult traits, respectively. PCA3 and PCA4 include only female data, with and without larval traits, respectively. For the other principal components see Table 4 (PCA 2) and Appendix S2

	PCA1	PCA2	PCA3	PCA4
Eigenvalue	**2.97**	**2.87**	**2.58**	**1.97**
Proportion of variance	**0.25**	**0.36**	**0.16**	**0.25**
Larval and pupal development				
Fifth instar weight	−0.274	−0.308	−0.369	–
Sixth instar weight	−0.121	−0.186	−0.230	–
Seventh instar weight	−0.023	−0.034	0.227	–
Fifth instar period	0.378	0.359	0.291	–
Sixth instar period	0.325	0.344	**0.502**	–
Seventh instar period	0.374	**0.403**	−0.082	–
Pupal weight	0.386	**0.409**	0.104	–
Pupal period	**−0.522**	**−0.541**	−0.383	–
Adult mobility and behaviors				
Longevity	−0.055	–	−0.125	**−0.503**
Mobility	−0.174	–	0.246	**0.436**
Probability of flying (transformed)	−0.217	–	−0.082	0.066
Probability of not moving (transformed)	0.140	–	–	–
Female reproductive traits				
Mating rate	–	–	−0.048	0.206
Lifetime-corrected number of eggs	–	–	0.233	0.365
Age at first oviposition	–	–	−0.335	−0.354
Mean clutch size	–	–	0.069	−0.162
Average time between two ovipositions	–	–	−0.047	**−0.475**
Average values				
Male	−1.127	−1.047	–	–
Female	1.241	1.172	–	–
Fragmented	−0.667	−0.735	−0.985	−0.782
Continuous	0.496	0.543	0.657	0.481
ANOVAs				
Sex	*P*<2.2e-16	*P*<2.2e-16	NA	NA
Landscape	*P=*6.87e-08	*P=*1.93e-09	*P=*0.00078	*P=*0.0040
Population	*P*=0.036	*P*=0.014	NS	NS
Sex × Population	*P=*2.73e-04	*P=*8.40e-05	NS	NS

Highest values from each PCA appear in bold for easier visualization of the results.

**Table 4:** Principal component analyses for male and female larval traits (G0-2009). Sex and landscape types explain much of the variation (see Fig. 6)

	PC1	PC2	PC3	PC4
Eigenvalue	**2.87**	**1.59**	**1.16**	**1.14**
Cumulative proportion of variance	**0.36**	**0.56**	**0.74**	**0.88**
Larval and pupal development				
Fifth instar weight	−0.308	−0.235	**−0.538**	0.146
Sixth instar weight	−0.186	−0.214	**−0.430**	**−0.669**
Seventh instar weight	−0.034	**−0.733**	0.132	−0.080
Fifth instar period	0.359	0.094	0.216	**−0.647**
Sixth instar period	0.344	**−0.476**	0.271	0.226
Seventh instar period	**0.403**	0.237	**−0.475**	0.162
Pupal weight	**0.409**	−0.263	−0.386	0.141
Pupal period	**−0.541**	−0.010	0.117	0.094
Average values				
Male	−1.047	0.210	0.506	−0.028
Female	1.172	−0.264	−0.593	0.031
Fragmented	−0.735	−0.223	−0.466	0.071
Continuous	0.543	0.165	0.344	−0.053
ANOVAs				
Sex	*P*<2.2e-16	*P=*0.019	*P=*2.12e-10	NS
Landscape	*P=*1.93e-09	*P*=0.048	*P=*3.001e-07	NS
Population	*P*=0.014	NS	NS	NS
Sex × Population	*P=*8.40e-05	NS	NS	NS

Highest values for each PCs appear in bold for easier visualization of the results.

In the analyses of female traits (PCA3 and PCA4), there are significant differences between the landscape types on PC1 (Table 3). Examining the coefficients in PCA4 suggests a syndrome of slow rate of oviposition but long life span in FL butterflies. The lifetime-corrected number of eggs laid is positively correlated with PC1, which has higher values in CL populations (Appendix S2, Table B).

## Discussion

We detected significant differences between fragmented versus CL in 17 of 51 larval and adult life-history traits, indicating that apparent landscape effects on the life history of the Glanville fritillary are common. The traits that were affected by landscape type mostly fall into three groups, namely measures of FMR, larval growth and development, and adult reproduction. We discuss these groups of traits in turn below.

### Habitat fragmentation, flight capacity, and dispersal

Previous studies on the Glanville fritillary in the Åland Islands have shown that female butterflies from newly-established populations have higher FMR (Haag et al. 2005; Wheat et al. 2011) and higher dispersal rate in the field (Ovaskainen et al. 2008) than butterflies from old local populations. In the large network of ∼4000 meadows, around 100 new local populations are established by dispersing females in every year (Hanski 2011), demonstrating a very high rate of population turnover. Given that FMR and dispersal rate have high heritability (Saastamoinen 2008), it is apparent that colonizers of previously unoccupied habitat patches are more dispersive butterflies than the average butterfly in the metapopulation, and hence, fast population turnover selects for increased rate of dispersal at the level of new populations. From this observation alone one cannot infer the network-level and long-term consequences of natural selection on dispersal, because the outcome at the landscape level depends on many other factors apart from selection at colonization (Ronce and Olivieri 2004; Ronce 2007). Nonetheless, three somewhat dissimilar models parameterized for the Glanville fritillary all predict that increasing fragmentation selects for increasing dispersal also at the network level (Heino and Hanski 2001; Zheng et al. 2009; Hanski and Mononen 2011). Given that there is a strong relationship between FMR and dispersal rate in the field (Niitepõld et al. 2009), the present results on FMR of butterflies from two separate fragmented (FL) and two separate CL strongly support the predicted higher dispersal rate in the former. Furthermore, relative thorax size was greater, and the wing loading ratio was smaller in FL than CL butterflies, indicating greater allocation to flight capacity in the former (Taylor and Merriam 1995; Van Dyck and Matthysen 1999; Karlsson and Van Dyck 2005; Karlsson and Johansson 2008). Our measures of mobility and movement distance in the outdoor population cage did not show systematic differences between the landscape types, but it is questionable whether butterfly movements within the cage, large as it is (∼900 m^2^), should correlate with between-population dispersal in the field. Finally, we observe that while the difference in the FMR between the landscape types is predicted by the difference in habitat fragmentation, the slight difference in latitude and therefore possibly in climatic conditions between the two landscape types does not lead to a plausible explanation of the difference in FMR.

Habitat fragmentation may have dissimilar effects on dispersal rate in different species depending on their ecologies and life histories. In many cases, high cost of dispersal, such as high mortality while moving across unsuitable matrix habitat, may select for reduced dispersal in fragmented habitats, which is the most common generalization about dispersal evolution in FL (Van Dyck and Baguette 2005; Baguette and Van Dyck 2007; Dytham and Travis 2012). For instance, several studies have reported that small mammals in fragmented habitats show reduced movements compared with populations inhabiting continuous habitat (Debinski and Holt 2000; Mora et al. 2010).

A particularly telling example is the contrast between the Glanville fritillary and the bog fritillary butterfly studied by Schtickzelle et al. (2006). Using mark–release–recapture experiments in four landscapes with dissimilar degree of fragmentation, they found that dispersal rate was lowest in the most fragmented landscape, in contrast to our results. An important difference between the two butterfly study systems is the much greater temporal stability of the local populations in the bog fritillary. Thus, Baguette (2004) found no population turnover at all in the bog fritillary in small networks of 16–20 patches that remained continuously occupied for 12 years, whereas similar small networks would not have viable metapopulations in the Glanville fritillary because of high rate of population turnover (Hanski and Ovaskainen 2000). Hanski (2011) suggested that the difference in the stability of local populations in the two butterfly species is due to greater risk-spreading by bog fritillary females, which lay many small clutches of 2–20 eggs (Radchuk et al. 2013) in contrast to a few large clutches of 150–200 eggs laid by Glanville fritillary. Whether this is the correct explanation or not for the dissimilar temporal stability of local populations in the two species, the results highlight the association between population turnover, availability of unoccupied habitat for colonization, and the direction of dispersal evolution in FL (for an extensive review of dispersal costs see Bonte et al. 2012).

Given that habitat fragmentation selects for increased dispersal, and thus higher colonization rate, in the Glanville fritillary, one may ask whether increased dispersal also enhances metapopulation viability in increasingly FL? Modeling results demonstrated such a possibility but only for a narrow range of parameter values (Heino and Hanski 2001), and it is known empirically that the Glanville fritillary has gone regionally extinct in many parts of its range due to habitat loss and fragmentation (Hanski et al. 1995; Kuussaari et al. 1995). One reason for the limited effect of increased dispersal on metapopulation viability is the direct adverse demographic effect of habitat loss and fragmentation, which is only partly compensated for by microevolutionary changes in dispersal rate. This is likely to be a common situation in eco-evolutionary dynamics in changing environments (Ellner et al. 2011). Additionally, habitat fragmentation in the real world may be associated with other adverse environmental changes, such as reduced density of host plants or reduced encounter rate of individuals with their resources (Morris 2003), which would further reduce metapopulation viability in FL.

### Larval development

Resources accumulated during the larval stage are essential for butterfly fitness. Adult butterflies use their body reserves for somatic maintenance and reproduction, as shown by large reduction in thorax (as well as abdomen) weight with aging, especially in reproducing females (Stjernholm and Karlsson 2000; Stjernholm et al. 2005; Saastamoinen et al. 2009). In the present study, we found a large difference in the postdiapause larval weight, which was 28% greater in FL than CL, and significantly faster postdiapause larval development in the former, while there was no difference in the pupal weight. Thus, larvae in FL started larger in the spring and grew faster, but to the same final size as larvae in CL.

Postdiapause larvae forage on new growing leaves. In the early spring, the larvae often consume all available leaves on a plant to the ground level. The larvae cannot afford waiting for plant recovery but disperse to forage within larger areas, up to several 10 m^2^ (Kuussaari et al. 1995). With low food availability in the spring, it may be advantageous to have a greater postdiapause body size and to grow fast when able to feed. One possible explanation of the dissimilar pattern of postdiapause growth between the landscape types relates to the more northern latitude of the two FL than the two CL (Fig. 1A). Although there is no systematic difference in the average temperatures (Fig. 1B), the host plant availability in the spring may nonetheless be slightly lower, at least in some years, in the slightly more northern localities (ÅL and UP). On the other hand, habitat fragmentation is also likely to influence food availability. In FL, dispersing females often encounter very small habitat patches with small numbers of host plants (Ojanen et al. 2013). In such situations, it would be advantageous for the larvae to have a large initial body size following winter diapause. In contrast, in CL with large expanses of suitable habitat, food availability is not likely to be similarly limiting.

There is extensive heritable and plastic variation in gene expression during postdiapause larval development in the Glanville fritillary (Kvist et al. 2013). In particular, larval serum protein (LSP) genes and cuticle-binding protein genes showed large and significant variation among larval families (indicative of heritability) and temperature treatments (Kvist et al. 2013). LSP are expressed in the fat body and secreted into the hemolymph during the final larval instar (Haunerland 1996) to be used as amino acid reservoirs in later development. Kvist et al. (2013) demonstrated that LSP expression was closely associated with larval development. Intriguingly, another study found a significant difference in LSP expression between female butterflies from newly established versus old local populations in Åland (Wheat et al. 2011). It is thus possible that the known difference between newly established and old local populations in FMR and dispersal (Hanski 2012a) is related, via shared molecular mechanisms, to fast postdiapause larval development. Our results provide further support for such phenotypic association at the landscape level, as butterflies in FL showed both fast postdiapause larval development and high FMR.

### Reproduction and longevity

The male reproductive success is primarily determined by the number of matings. In the present experiment, males from FL had significantly higher frequency of multiple matings than expected by chance and higher than males from CL. This result probably reflects the greater flight metabolism (Fig. 5A) and flight activity (Fig. 5B) of FL than CL males. Males from ÅL exhibited the highest flight metabolism (Fig. 5A), and they also had the highest mating success in competition with males from the other populations in the outdoor cage (Table 2). Additionally, ÅL butterflies showed a significant tendency toward assortative mating (Table 2), which may reflect some behavioral differences, such as variation in courtship signals, between the four populations.

In highly FL, such as the Åland Islands, local populations are often small and consist mostly of full sibs (Saccheri et al. 1998; Austin et al. 2011). In such situations, females that mate several times would increase their chances of encountering unrelated partners and therefore reduce the risk of producing only inbred offspring (Sarhan and Kokko 2007). In our experiment, 12 of 80 females mated twice in the outdoor cage, with no difference between females from the two landscape types (Appendix S1, Table A1). Of the 12 polyandrous females, eight had first mated with a nonvirgin male, while out of the 62 monandrous females, only 13 mated with a nonvirgin male (Fisher's exact test, *P* = 0.003). Males' ejaculate (spermatophore) decreases in size and quality following previous matings (Svärd and Wiklund 1986), and hence, females may seek for another mate when their first mate's investment (spermatophore) was small (Charlat et al. 2007). The four polyandrous females that mated for the second time after first having mated with a virgin male were all from FL (Fisher's exact test, *P* *=* 0.03; numbers of females from Table 1). This result may reflect within-generation bet hedging to avoid adverse consequences of inbreeding in the often small populations in FL as suggested by Sarhan and Kokko (2007).

Gibbs and Van Dyck (2010) have shown that the energetic cost of dispersal reduces longevity and affects negatively resource allocation to egg production in the specked wood butterfly (*P. aegeria*). In our analysis (PCA4 in Table 3), females with shorter intervals between consecutive egg clutches had shorter longevity, and these females also exhibited greater mobility in the cage, perhaps reflecting searching for oviposition host plants within populations. There was a significant difference between the landscape types, as females from CL showed higher mobility in the cage, shorter interval between ovipositions, somewhat higher life-time egg production, but shorter longevity. Females from FL that frequently disperse from one habitat patch to another may often have no immediate opportunity to lay the next egg clutch, and hence, selection may favor lower rate of egg maturation than in CL females. Dispersive females need energy to fuel flight, which may further reduce the rate of egg maturation.

## Conclusion

We have documented multiple life-history differences between butterflies originating from fragmented versus CL especially in FMR (influencing movements and dispersal), larval growth and development, and adult reproduction. All differences can be explained parsimoniously by the typically small size and high turnover rate of local populations in FL. Models predict that high turnover of small populations selects for increased flight capacity and dispersal, and low food availability in the spring in the small habitat patches may select for increased size of diapausing larvae in FL. Females from CL exhibited higher rate of ovipositing egg clutches but shorter longevity than females from FL. We conclude that habitat fragmentation has significant consequences for the life history of the Glanville fritillary.

## Supporting Information

**Appendix S1.** Tables describing the variables, mean values and the *P*-values for all statistical tests.

**Appendix S2.** Principal component analyses.
