# Supplementary material for: Life history of the Glanville fritillary butterfly in fragmented versus continuous landscapes
Source: Ecol Evol. 2013 Nov 22;3(16):5141–56. doi: 10.1002/ece3.885 (PMC3892324; doi:10.1002/ece3.885)
Supplement: Supplementary file 1 [file ece30003-5141-SD1.doc]

Appendix - A. Tables describing the variables, mean values and the *P*-values for all statistical tests.

**Table A1. Average values for each population and both sexes, and the significance of the difference between the landscape types.**

| **Landscape** | | **Fragmented (FL)** | | | | **Continuous (CL)** | | | | Significant landscape difference |
| --- | --- | --- | --- | --- | --- | --- | --- | --- | --- | --- |
| Population | | Åland (ÅL) | | Uppland (UP) | | Öland (ÖL) | | Saaremaa (SA) | |
| Sex | | **♂** | **♀** | **♂** | **♀** | **♂** | **♀** | **♂** | **♀** |
| ***Larval and pupal development*** | | | | | | | | | | |
|  | 5^th^ instar weight (mg) | 5.06 | 5.37 | 5.65 | 5.44 | 4.58 | 3.83 | 3.48 | 3.83 | FL > CL *** |
|  | 6^th^ instar weight (mg) | 21.12 | 24.41 | 20.45 | 19.92 | 19.04 | 18.05 | 17.10 | 18.62 | FL > CL *** |
|  | 7^th^ instar weight (mg) | 72.09 | 71.07 | 64.67 | 66.18 | 66.51 | 71.45 | 63.05 | 68.25 | NS |
|  | 5^th^ instar period (days) | 6.90 | 7.57 | 7.38 | 7.67 | 7.42 | 8.5 | 8.44 | 8.43 | FL < CL *** |
|  | 6^th^ instar period (days) | 5.7 | 6.29 | 5.83 | 6.67 | 5.84 | 6.94 | 6.12 | 6.39 | NS |
|  | 7^th^ instar period (days) | 7.92 | 10.15 | 7.79 | 9.17 | 9.47 | 11.69 | 8.58 | 10.22 | NS |
|  | Post-diapause larval period (days) | 20.16 | 23.63 | 20.36 | 22.41 | 22.74 | 27.13 | 22.10 | 24.58 | FL < CL *** |
|  | Larval weight gain (mg) | 31.63 | 37.03 | 27.89 | 35.81 | 34.62 | 52.10 | 48.14 | 50.78 | FL < CL *** |
|  | Relative growth rate (RGR) | 1.43 | 1.45 | 1.25 | 1.41 | 1.51 | 1.93 | 1.97 | 1.96 | FL < CL *** |
|  | Pupal weight (mg) (G0) | 153.09 | 189.64 | 149.12 | 191.14 | 150.05 | 194.16 | 156.67 | 194.00 | NS |
|  | Pupal weight (mg) (G1) | 137.33 | 153.48 | 129.55 | 161.15 | 143.21 | 168.26 | 143.11 | 176.22 | NS |
|  | Pupal period (days) | 16.81 | 14.00 | 15.33 | 12.39 | 15.74 | 12.38 | 14.19 | 13.10 | FL > CL ** |
| ***Longevity and Reproduction*** | | | | | | | | | | |
|  | Longevity | 5.90 | 6.21 | 6.46 | 5.83 | 5.16 | 5.56 | 5.74 | 5.68 | NS |
|  | Mating rate | 0.26 | 0.14 | 0.15 | 0.21 | 0.16 | 0.18 | 0.08 | 0.24 | NS |
|  | Female’s number of matings | - | 0.93 | - | 1.17 | - | 0.81 | - | 1 | NS |
|  | Age at 1st mating (days) | 1.25 | 0.91 | 1.27 | 1.56 | 1.1 | 0.83 | 1.69 | 0.93 | NS |
|  | Age at 1st oviposition (days) | - | 3.00 | - | 4.00 | - | 3.27 | - | 2.76 | FL > CL * |
|  | Oviposition rate (*ArcSin*) | - | 0.040 | - | 0.042 | - | 0.054 | - | 0.045 | NS |
|  | Lifetime number of eggs | - | 32.30 | - | 35.30 | - | 58.45 | - | 34.30 | NS |
|  | Clutch size (weight corrected) | - | 0.72 | - | 0.70 | - | 0.74 | - | 0.60 | NS |
|  | 1st clutch size (weight corrected) | - | 0.86 | - | 0.71 | - | 0.83 | - | 0.62 | NS |
|  | Length of oviposition (min) | - | 43.29 | - | 53.29 | - | 47.12 | - | 50.74 | NS |
|  | Length of 1st oviposition (min) | - | 55.77 | - | 62.18 | - | 49 | - | 68.2 | NS |
|  | Oviposition speed (eggs/min) | - | 0.43 | - | 0.62 | - | 0.33 | - | 0.47 | NS |
|  | 1st oviposition speed (eggs/min) | - | 4.89 | - | 3.66 | - | 4.09 | - | 2.77 | NS |
|  | Time between 2 clutches (days) | - | 3.43 | - | 3.77 | - | 3 | - | 2.25 | FL > CL *** |
|  | Time between 1^st^ and 2^sd^ clutches | - | 2.78 | - | 2.75 | - | 1.8 | - | 1.53 | FL > CL * |
|  | Hatch rate (*ArcSin*) | - | 1.22 | - | 1.05 | - | 1.15 | - | 1.17 | NS |
|  | 1st clutch hatch rate (*ArcSin*) | - | 1.19 | - | 0.77 | - | 1.06 | - | 1.08 | NS |
|  | Egg weight (mg) | - | 0.088 | - | 0.074 | - | 0.095 | - | 0.084 | NS |
|  | Preference for Plantago (*ArcSin*) | - | 0.054 | - | 0.068 | - | 0.048 | - | 0.0053 | FL > CL *** |
| ***Behavior, metabolism and morphology*** | | | | | | | | | | |
|  | Mobility | 1.39 | -0.78 | 0.86 | -0.25 | 0.17 | 0.03 | 1.59 | 0.51 | NS |
|  | Young butterflies' mobility | 0.35 | -0.82 | -0.29 | -0.48 | 0.12 | -0.16 | 0.41 | 0.34 | FL < CL * |
|  | Dispersal | 0.048 | -0.30 | -0.014 | -0.32 | 0.22 | 0.18 | -0.036 | 0.10 | NS |
|  | Distance (m) | 3.95 | 3.67 | 3.75 | 3.62 | 4.14 | 3.99 | 3.91 | 4.04 | NS |
|  | Resting (*ArcSin*) | 0.456 | 0.674 | 0.482 | 0.598 | 0.553 | 0.614 | 0.619 | 0.601 | NS |
|  | Basking (*ArcSin*) | 0.221 | 0.270 | 0.292 | 0.289 | 0.296 | 0.238 | 0.233 | 0.210 | NS |
|  | No movement (*ArcSin*) | 0.737 | 1.093 | 0.905 | 0.981 | 0.978 | 0.900 | 0.996 | 0.820 | NS |
|  | Flying (*ArcSin*) | 0.231 | 0.113 | 0.165 | 0.104 | 0.149 | 0.152 | 0.159 | 0.155 | NS |
|  | FMR peak CO_2_ (weight corrected) | 0.15 | 0.089 | 0.11 | 0.068 | 0.039 | 0.032 | 0.12 | 0.085 | FL > CL *** |
|  | FMR Int CO_2_ (weight corrected) | 0.018 | 0.011 | 0.013 | 0.0083 | 0.0038 | 0.0038 | 0.012 | 0.010 | FL > CL *** |
|  | Endurance (weight corrected) | 0.0084 | 0.0053 | 0.0059 | 0.0041 | 0.0018 | 0.0021 | 0.0051 | 0.0047 | FL > CL ** |
|  | RMR (weight corrected) | 0.0051 | 0.0052 | 0.0067 | 0.0038 | 0.0020 | 0.0019 | 0.0062 | 0.0047 | NS |
|  | Abdomen dry weight (mg) | 9.06 | 13.37 | 8.33 | 15.93 | 10.62 | 16.96 | 11.32 | 18.60 | NS |
|  | Thorax dry weight (mg) | 7.38 | 7.86 | 6.49 | 8.32 | 7.84 | 8.94 | 7.30 | 9.19 | NS |
|  | Total dry weight (mg) | 16.44 | 21.22 | 14.81 | 24.98 | 18.46 | 25.90 | 18.62 | 27.79 | NS |
|  | Thorax body allocation | 0.45 | 0.38 | 0.45 | 0.34 | 0.43 | 0.35 | 0.40 | 0.33 | NS |
|  | Forewing large area (mm^2^) | 54.74 | 65.14 | 53.00 | 67.83 | 56.96 | 67.44 | 56.32 | 73.87 | NS |
|  | Forewing small area (mm^2^) | 17.06 | 20.69 | 16.96 | 21.93 | 18.38 | 20.95 | 17.77 | 23.14 | NS |
|  | Thorax wing load (mg/mm^2^) | 0.13 | 0.12 | 0.12 | 0.12 | 0.14 | 0.13 | 0.13 | 0.12 | NS |
|  | Wing load (mg/mm^2^) | 0.30 | 0.33 | 0.28 | 0.36 | 0.32 | 0.38 | 0.33 | 0.37 | FL < CL * |

**Table A2. Details of statistical tests on the traits with significant differences and traits mentioned in the paper.**

| Trait | | **Material** | **Statistics** | **Landscape** | **Population** | **Family** | **Sex** | **Other factors** |
| --- | --- | --- | --- | --- | --- | --- | --- | --- |
| ***Larval and pupal development*** | | | | | | | | |
|  | 5^th^ instars weight (mg) | G0-2009 | ANOVA | *F=*53.13, df=1, *P=*1.30e-11 | NS | - | NS | - |
|  | 6^th^ instars weight (mg) | G0-2009 | ANOVA | *F=*17.52, df=1, *P=*4.65e-5 | NS | - | NS | - |
|  | 7^th^ instars weight (mg) | G0-2006 + 2009 | ANOVA | NS | *F=*3.29, df=2, *P=*0.039 | *-* | NS | Year: NS |
|  | 5^th^ instars period (mg) | G0-2009 | ANOVA | *F=*35.68, df=1, *P=*1.71e-08 | *F=*7.44, df=2, *P=*8.39e-04 | - | *F=*14.31, df=1, *P=*2.26e-04 | - |
|  | Post-diapause larval period (days) | G0-2006 + 2009 | ANOVA | *F*=23.88, df=1, *P*=1.79e-06 | NS | *-* | *F*=174.70, df=1, *P<*2.2e-16 | Year: *F=*350.73,df=1, *P<*2.2e-16 |
|  | Larval weight gain | G0-2009 | ANOVA | *F=*65.20, df=1, *P=*1.67e-13 | *F*=*=*4.25, df=2, *P=*0.016 | - | *F=*28.84, df=1, *P*=2.78e-7 | - |
|  | Relative Growth Rate (RGR) | G0-2009 | ANOVA | *F*=53.92, df=1, *P*=1.003e-11 | *F*=4.96, df=2, *P*=0.0081 | - | *F*=7.46, df=1, *P*=0.0070 | - |
|  | Pupal weight (mg) | G0-2009 | ANOVA | NS | NS | - | *F=*322.51, d=1*, P<*2.2e-16 | - |
|  | Pupal weight (mg) | G1-2009 | Mixed model | NS | NS | NS | *F=*55.28*,*dfn=1*, P*<0.0001 | - |
|  | Pupal period (days) | G0-2009 | ANOVA | *F=*10.04*,* d=1, P=0.0018 | *F=*10.22, df=2, *P=*6.59e-5 | *-* | *F=*106.72, d=1, *P<*2.2e-16 | - |
| ***Longevity and Reproduction*** | | | | | | | | |
|  | Longevity | G0-2009 | G.L.M. | NS | NS | - | NS | - |
|  | Age at 1st mating (*log*) | G0-2009 | ANOVA | NS | NS | - | *F*=4.87, df=1, *P*=0.029 | - |
|  | Age at 1st oviposition (*log*) | G0-2009 | ANOVA | *F=*6.68, df=1, *P=*0.013 | NS | - | - | - |
|  | Oviposition rate (*ArcSin*) | G0-2009 | ANOVA | NS | NS | - | - | - |
|  | Lifetime number of eggs | G0-2009 | ANOVA | NS | NS | - | - | Pupal weight: *F=*11.25, df=1, *P=*0.0013 |
|  | Clutch size (weight corrected) | G0-2009 | ANOVA | NS | NS | *-* | - | Clutch rank: *F=*7.57, df=1, *P=*0.0065 |
|  | 1^st^ clutch size  (weight corrected) | G0-2009 | ANOVA | NS | NS | - | - | - |
|  | 1^st^ oviposition length | G0-2009 | ANOVA | NS | NS | - | - | Clutch rank: NS |
|  | 1^st^ oviposition speed (*log*) | G0-2009 | ANOVA | NS | NS | - | - | - |
|  | Time between two clutches | G0-2009 | ANOVA | *F=*17.13, df=1, *P=*6.17e-05 | NS | - | - | Clutch size: *F=*55.09, df=1, *P=*1.23e-11  Clutch rank: df=1, *F=*19.84, *P=*1.77e-05 |
|  | Time between 1^st^ clutches | G0-2009 | ANOVA | *F=*6.12, df=1, *P=*0.018 | NS | - | - | Clutch size: *F=*19.71, df=1, *P=*6.67e-05 |
|  | 1^st^ clutch hatch rate (*ArcSin*) | G0-2009 | ANOVA | NS | NS | - | - | Clutch size: *F=*22.25, df=1, *P=*2.17e-05 |
|  | Preference for Plantago (*ArcSin*) | G0-2009 | ANOVA | *F=*26.06, df=1, *P=*4.76e-06 | *F=*8.66, df=2, *P=*5.67e-05 | - | - | - |
| ***Behavior, metabolism and morphology*** | | | | | | | | |
|  | Mobility | G0-2009 | ANOVA | NS | NS | - | *F=*15.69, df=1, *P=*1.11e-4 | - |
|  | Young butterflies' mobility | G0-2009 | ANOVA | *F=*4.92, df=1, *P=*0.028 | NS | *-* | NS | - |
|  | Resting (*ArcSin*) | G0-2009 | ANOVA | NS | NS | - | NS | - |
|  | Flying (*ArcSin*) | G0-2009 | ANOVA | NS | NS | - | *F=*4.22*,* df=1*, Padj*=0.042 | Sex:Landscape: *Padj*=0.016 |
|  | FMR peak CO_2_  (weight corrected) | G1-2009 | Mixed model | *F=*16.21*,* dfn=1*, P*=9.0e-04 | NS | NS | *F=*14.64*,* dfn=1*, P*=4.00e-04 | - |
|  | FMR Int CO_2_  (weight corrected) | G1-2009 | Mixed model | *F=*20.37*,* dfn=1*, P*=3.0e-04 | NS | NS | *F=*4.27*,* dfn=1*, P*=0.044 | - |
|  | Endurance (weight corrected) | G1-2009 | Mixed model | *F=*10.64*,* dfn=1*, P*=0.0046 | NS | NS | NS | - |
|  | RMR (weight corrected) | G1-2009 | Mixed model | NS | NS | NS | NS | - |
|  | Abdomen dry weight | G1-2009 | Mixed model | NS | NS | NS | *F=*115.1*,* dfn=1*, P*<0.0001 | - |
|  | Thorax dry weight | G1-2009 | Mixed model | NS | NS | NS | *F=*27.80*,* dfn=1*, P*<0.0001 | - |
|  | Adult dry weight | G1-2009 | Mixed model | NS | NS | NS | *F=*96.63*,* dfn=1*, P*<0.0001 | - |
|  | Forewing large area | G1-2009 | Mixed model | NS | NS | NS | *F=*146.4*,* dfn=1*, P*<0.0001 | - |
|  | Wing load | G1-2009 | Mixed model | *F=*20.33*,* dfn=1*, P*=0.046 | NS | NS | *F=*25.73*,* dfn=1*, P*<0.0001 | - |

Appendix - B. Principal component analyses.

Table B1. PCA1 for male and female larval and adult traits.

|  | **PC1** | **PC2** | **PC3** | **PC4** | **PC5** |
| --- | --- | --- | --- | --- | --- |
| **Eigenvalues** | 2.969 | 1.940 | 1.600 | 1.488 | 1.138 |
| **Cumulative proportion** | 0.25 | 0.41 | 0.54 | 0.67 | 0.76 |
|  | | | | | |
| ***Larval and pupal development*** | | | | | |
| 5^th^ instar weight | -0.274 | -0.261 | 0.080 | **-0.515** | 0.176 |
| 6^th^ instar weight | -0.121 | -0.335 | 0.017 | **-0.419** | **-0.525** |
| 7^th^ instar weight | -0.023 | -0.092 | **0.719** | -0.070 | -0.195 |
| 5^th^ instar period | 0.378 | -0.024 | -0.094 | 0.247 | **-0.514** |
| 6^th^ instar period | 0.325 | 0.120 | **0.536** | 0.114 | 0.091 |
| 7^th^ instar period | 0.374 | 0.118 | -0.328 | **-0.406** | 0.200 |
| Pupal weight | 0.386 | 0.060 | 0.184 | **-0.452** | 0.119 |
| Pupal period | **-0.522** | -0.105 | 0.0370 | 0.095 | 0.049 |
| ***Adult mobility and behaviors*** | | | | | |
| Longevity | -0.055 | 0.290 | 0.066 | -0.199 | 0.130 |
| Mobility | -0.174 | 0.341 | 0.180 | 0.071 | 0.316 |
| Probability of flying (*ArcSin*) | -0.217 | **0.542** | -0.011 | -0.110 | -0.282 |
| Probability of not moving (*ArcSin*) | 0.140 | **-0.523** | 0.032 | 0.211 | 0.362 |
|  | | | | | |
| ***Average values*** |  |  |  |  |  |
| Male | -1.127 | -0.036 | -0.039 | 0.575 | -0.034 |
| Female | 1.241 | 0.049 | 0.068 | -0.671 | 0.036 |
| Fragmented | -0.667 | -0.257 | 0.074 | -0.535 | 0.085 |
| Continuous | 0.496 | 0.192 | -0.055 | 0.398 | -0.063 |
| ***ANOVAs*** | | | | | |
| Sex | *P* < 2.2e-16 | NS | NS | *P=*1.34e-13 | NS |
| Landscape | *P=*6.87e-08 | *P*=0.044 | NS | *P=* 1.53e-09 | NS |
| Population | *P*=0.036 | NS | NS | NS | NS |
| Interaction | Sex:Pop -*P=*2.73e-4 | Sex:Land -*P*=0.019 | NS | NS | NS |

Table B2. PCA3 for female larval and adult traits.

|  | | | | **PC1** | | | | **PC2** | | | | **PC3** | | | | **PC4** | | | **PC5** | | **PC6** | | **PC7** |
| --- | --- | --- | --- | --- | --- | --- | --- | --- | --- | --- | --- | --- | --- | --- | --- | --- | --- | --- | --- | --- | --- | --- | --- |
| **Eigenvalues** | | | | 2.579 | | | | 2.202 | | | | 2.059 | | | | 1.796 | | | 1.544 | | 1.384 | | 1.031 |
| **Cumulative proportion** | | | | 0.16 | | | | 0.30 | | | | 0.43 | | | | 0.54 | | | 0.64 | | 0.72 | | 0.79 |
|  | | | | | | | | | | | | | | | | | | | | | | | |
| ***Larval and pupal development*** | | | | | | | | | | | | | | | | | | | | | | | |
| 5^th^ instar weight | -0.369 | | | | **0.410** | | | | -0.040 | | | | 0.072 | | | | -0.123 | | | 0.213 | | -0.059 | |
| 6^th^ instar weight | -0.230 | | | | 0.020 | | | | -0.290 | | | | 0.430 | | | | -0.059 | | | 0.339 | | -0.184 | |
| 7^th^ instar weight | 0.227 | | | | 0.362 | | | | -0.114 | | | | 0.200 | | | | 0.156 | | | **0.401** | | 0.030 | |
| 5^th^ instar period | 0.291 | | | | -0.370 | | | | -0.180 | | | | 0.371 | | | | 0.115 | | | -0.009 | | -0.015 | |
| 6^th^ instar period | **0.502** | | | | 0.142 | | | | -0.105 | | | | 0.070 | | | | 0.020 | | | 0.040 | | -0.125 | |
| 7^th^ instar period | -0.082 | | | | -0.298 | | | | 0.037 | | | | **-0.418** | | | | **-0.432** | | | 0.083 | | -0.072 | |
| Pupal weight | 0.104 | | | | 0.170 | | | | -0.197 | | | | -0.353 | | | | -0.272 | | | **0.431** | | -0.154 | |
| Pupal period | -0.383 | | | | 0.376 | | | | 0.084 | | | | 0.001 | | | | 0.193 | | | -0.183 | | 0.339 | |
| ***Adult mobility and behaviors*** | | | | | | | | | | | | | | | | | | | | | | | |
| Mobility | 0.246 | | | | 0.300 | | | | 0.330 | | | | -0.230 | | | | -0.005 | | | -0.002 | | -0.112 | |
| Longevity | -0.125 | | | | -0.149 | | | | -0.089 | | | | -0.291 | | | | **0.406** | | | 0.276 | | -0.325 | |
| Probability of flying (*ArcSin*) | -0.082 | | | | 0.100 | | | | 0.273 | | | | 0.032 | | | | 0.251 | | | -0.231 | | **-0.720** | |
| ***Female reproductive traits*** | | | | | | | | | | | | | | | | | | | | | | | |
| Mating rate | -0.048 | | | | 0.106 | | | | -0.308 | | | | 0.061 | | | | **-0.461** | | | -0.246 | | -0.120 | |
| Lifetime corrected number of eggs | 0.233 | | | | 0.310 | | | | -0.181 | | | | 0.062 | | | | -0.188 | | | **-0.430** | | -0.149 | |
| Age at 1^st^ Oviposition | -0.335 | | | | -0.137 | | | | -0.219 | | | | 0.129 | | | | -0.076 | | | -0.148 | | -0.313 | |
| Mean clutch size | 0.069 | | | | 0.179 | | | | **-0.480** | | | | -0.286 | | | | 0.202 | | | -0.184 | | -0.052 | |
| Average time between 2 ovipositions | -0.047 | | | | -0.064 | | | | **-0.461** | | | | -0.288 | | | | 0.352 | | | -0.143 | | 0.167 | |
|  | | | | | | | | | | | | | | | | | | | | | | | |
| ***Average values*** | | | | | | | | | | | | | | | | | | | | | | | |
| Fragmented | | | -0.985 | | | | 0.272 | | | | -0.676 | | | | -0.052 | | | -0.083 | | | -0.365 | | 0.239 |
| Continuous | | | 0.657 | | | | -0.181 | | | | 0.451 | | | | 0.035 | | | 0.056 | | | 0.243 | | -0.159 |
| ***ANOVAs*** | | | | | | | | | | | | | | | | | | | | | | | |
| Landscape | | *P=*7.8e-04 | | | | NS | | | | *P*=0.011 | | | | NS | | | | NS | | | NS | | NS |
| Population | | NS | | | | NS | | | | NS | | | | NS | | | | NS | | | NS | | NS |

Table B3. PCA4 for female adult.

|  | | **PC1** | | **PC2** | | **PC3** | **PC4** |
| --- | --- | --- | --- | --- | --- | --- | --- |
| **Eigenvalues** | | 1.966 | | 1.804 | | 1.293 | 1.084 |
| **Cumulative proportion** | | 0.246 | | 0.471 | | 0.633 | 0.768 |
|  | | | | | | | |
| ***Female mobility and behaviors*** | | | | | | | |
| Longevity | | **-0.503** | | 0.161 | | -0.335 | 0.014 |
| Mobility | | **0.436** | | 0.065 | | **-0.502** | -0.124 |
| Probability of flying (*ArcSin*) | | 0.066 | | 0.204 | | **-0.475** | **0.705** |
| ***Female reproductive traits*** | | | | | | | |
| Mating rate | | 0.206 | | **-0.460** | | 0.334 | 0.174 |
| Lifetime corrected number of eggs | | 0.365 | | **-0.462** | | -0.131 | 0.248 |
| Age at 1^st^ Oviposition | | -0.354 | | -0.054 | | 0.327 | **0.596** |
| Mean clutch size | | -0.162 | | -0.586 | | -0.328 | -0.014 |
| Average time between 2 ovipositions | | **-0.475** | | -0.396 | | -0.259 | -0.203 |
|  | | | | | | | |
| ***Average values*** | |  | |  | |  |  |
| Fragmented | | -0.782 | | -0.189 | | 0.229 | 0.232 |
| Continuous | | 0.481 | | 0.116 | | -0.141 | -0.143 |
| ***ANOVAs*** | | | | | | | |
| Landscape | *P=*0.0040 | | NS | | NS | | NS |
| Population | NS | | NS | | NS | | NS |
